# Advances in treatment strategies based on scavenging reactive oxygen species of nanoparticles for atherosclerosis

**DOI:** 10.1186/s12951-023-02058-z

**Published:** 2023-08-17

**Authors:** Chengxi Wu, Jingying Mao, Xueqin Wang, Ronghao Yang, Chenglong Wang, Chunhong Li, Xiangyu Zhou

**Affiliations:** 1 Department of Thyroid and Vascular Surgery, the Affiliated Hospital of Southwest Medical University, No. 25, Taiping Street, Luzhou, Sichuan 646000 China; 2 Department of Pharmaceutical Sciences, School of Pharmacy, Southwest Medical University, 1-1 Xianglin Road, Luzhou, Sichuan 646000 China; 3 Department of Thyroid Surgery, people’s Hospital of Deyang, Deyang, Sichuan 618000 China

**Keywords:** Nanoparticles, Scavenging ROS, Treatment strategies, Atherosclerosis

## Abstract

The development of atherosclerosis (AS) is closely linked to changes in the plaque microenvironment, which consists primarily of the cells that form plaque and the associated factors they secrete. The onset of inflammation, lipid deposition, and various pathological changes in cellular metabolism that accompany the plaque microenvironment will promote the development of AS. Numerous studies have shown that oxidative stress is an important condition that promotes AS. The accumulation of reactive oxygen species (ROS) is oxidative stress’s most important pathological change. In turn, the effects of ROS on the plaque microenvironment are complex and varied, and these effects are ultimately reflected in the promotion or inhibition of AS. This article reviews the effects of ROS on the microenvironment of atherosclerotic plaques and their impact on disease progression over the past five years and focuses on the progress of treatment strategies based on scavenging ROS of nanoparticles for AS. Finally, we also discuss the prospects and challenges of AS treatment.

## Introduction

Atherosclerosis (AS) is a chronic inflammatory disease induced by a variety of factors and is a major cause of cardiovascular disease. Recent studies have shown that AS is one of the leading causes of death in the elderly, and its incidence is increasing among young people, posing a serious threat to life and health. The development and progression of AS is a complex pathological process, and the pathogenesis includes the onset of inflammation, oxidative stress, and dysfunction of various cells, signaling molecules, and pathways [1, 2]. Alterations in the microenvironment of blood circulation, such as inflammation, lipid deposition, endothelial cell dysfunction, smooth muscle cell proliferation, and macrophage apoptosis, all accelerate the formation of AS [3–6]. Recent evidence suggests that oxidative stress is a key factor in the development of AS [7]. Oxidative stress occurs mainly as a result of an imbalance between the production of reactive oxygen species (ROS) and antioxidants [8]. ROS is a double-edged sword in the body. When reactive oxygen is at normal levels in the body, phagocytes can maintain the stability of the organism by performing normal phagocytosis. When the concentration of ROS is slightly elevated, it is considered to be a signal of replenishment in the cells, which facilitates cell proliferation, angiogenesis, and metastasis and has an early warning effect [9]. Under normal conditions, ROS are scavenged mainly through enzymatic or non-enzymatic mechanisms to maintain oxidative and antioxidant homeostasis in the body [10]. When the rate of production is greater than the rate of clearance, excess ROS accumulates in the body and the body tends to oxidize, producing more oxidative substances and some proteases, accompanied by neutrophil inflammatory infiltration, so ROS is closely related to inflammation and cell death, accelerating the development of disease [11–14].

## Sources of ROS

ROS refers to a group of chemically active oxygenated substances, mainly including superoxide anion (O_2_·^−^), hydroxyl radical (·OH), peroxyl radical (ROO·), hydrogen peroxide (H_2_O_2_), ozone (O_3_), and hypochlorous acid (HOCL) [15–18]. One type of reactive oxygen can be converted to another type of reactive oxygen through a series of reactions [19]. Thus, H_2_O_2_ is the main form of intracellular ROS present [20]. The intrinsic source of ROS is mainly produced by biochemical reactions during mitochondrial respiration, with about 90% of cellular ROS coming from mitochondria [21–23]. ROS are produced by various enzymes such as nicotinamide adenine dinucleotide phosphate (NADPH) oxidases (NOXs), xanthine oxidase (XOD), nitric oxide synthase (NOS) and peroxidase (POD), etc. [16, 18].

## Mechanisms of AS formation facilitated by ROS

Studies have shown that the initial stage of AS is mainly due to endothelial cell (ECs) dysfunction. The accumulation of ROS positively contributes to processes such as inflammation, ECs dysfunction, and macrophage infiltration, which all contribute to AS [24, 25]. With an excessive amount of ROS on the basement membrane of vascular ECs, the body’s antioxidant system will be impaired, which leads to a decrease in the activity of superoxide dismutase (SOD), catalase (CAT), glutathione-S-transferase (GST) and other antioxidant enzymes, resulting in impaired ECs barrier function[26]. At the same time, inflammation is an important driver of the development of AS [13, 27–29]. ROS can promote the expression of inflammatory factors such as vascular cellular adhesion molecule-1 (VCAM-1), interleukin 6 **(**IL-6), interleukin (IL)-1β, and other inflammatory factors by activating the NF-kappaB (NF-κB) pathway [14, 30, 31]. Inflammatory factors have a negative feedback effect on ECs, which further exacerbates ECs damage [32, 33]. In addition, inflammatory factors also promote the proliferation, adhesion, and migration of vascular smooth muscle cells (VSMCs) [34]. At the plaque, low-density lipoprotein (LDL) is oxidized to ox-LDL (Oxidized low-density lipoprotein) due to the large accumulation of ROS, and the uptake of ox-LDL by macrophages and VSMCs on the one hand promotes the development of lipid deposition [35]. On the other hand, ox-LDL has a direct cytotoxic effect on ECs [36, 37], the enlargement of interendothelial gaps and increased permeability of ECs allows lipid components to enter directly into the subendothelium resulting in lipid deposition [38–40]. All of these pathways will promote the formation of AS, and it is easy to see that the promotion of AS by ROS is multifaceted and interconnected, which opens up the possibility of treating AS in more directions.

### Current status of AS treatment based on nanodelivery systems

Currently, the treatment of atherosclerosis includes lipid regulation, antiplatelet therapy, and antihypertensive therapy. Among the regulation of lipids, statins are the cornerstone of pharmacological treatment for dyslipidemia and have an important role in preventing and controlling atherosclerotic cardiovascular disease at home and abroad [41, 42]. However, there are significant side effects in the current atherosclerosis drug regimen, such as hepatotoxicity of statins, which can cause rhabdomyolysis and myocardial infarction, and the uncontrollable risk of bleeding with antiplatelet drugs and thrombolytics [43, 44]. To reduce the adverse effects of drugs and improve the efficiency of treatment, it is important to explore new forms of drug delivery to intervene in AS.

**Fig. 1:**
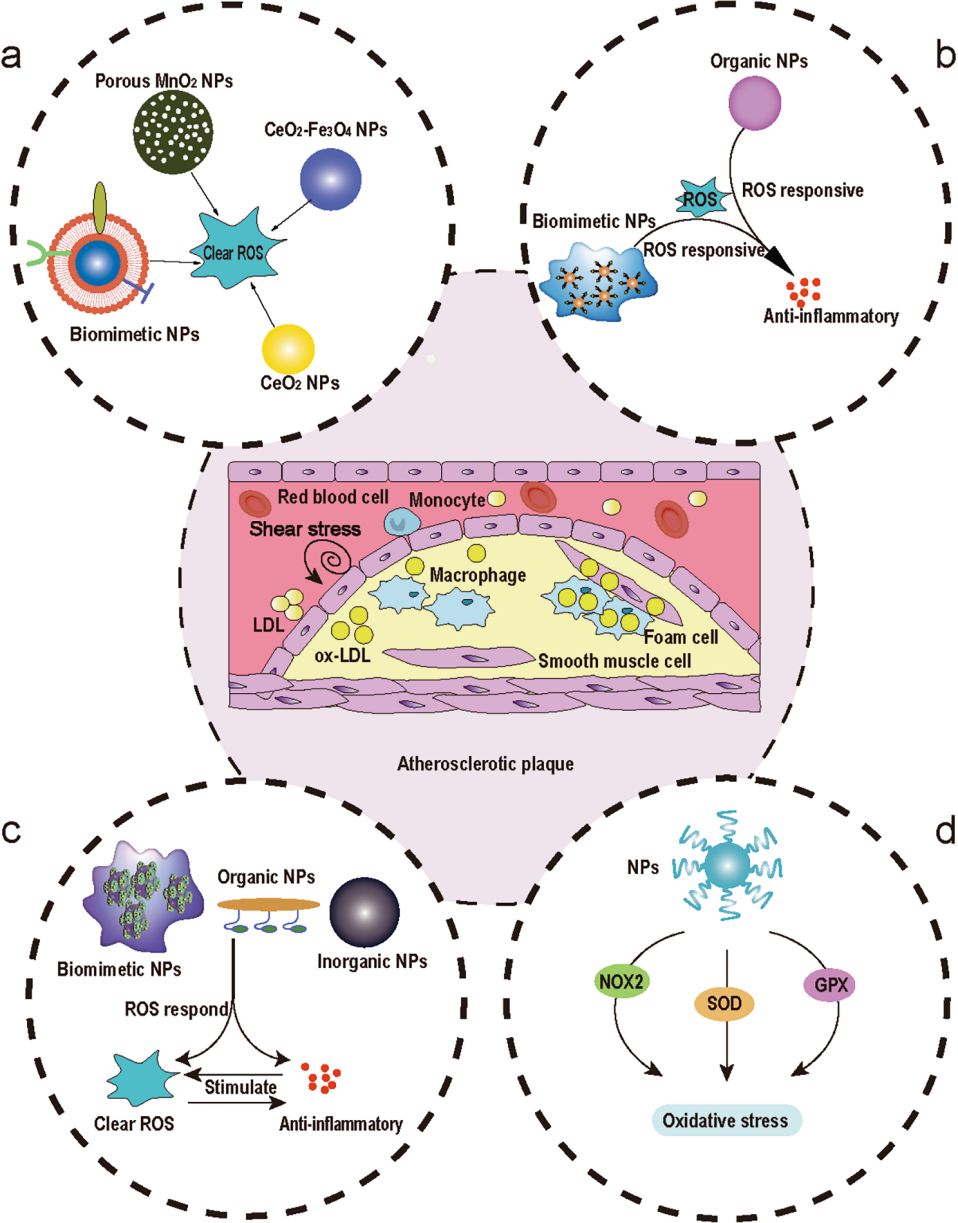
Schematic diagram of the atherosclerotic plaque microenvironment and treatment strategies based on NPs for AS. **a** ROS scavenging strategies. **b** ROS responsive strategies. **c** ROS responsive and ROS scavenging strategies. **d** Regulation of oxidative stress strategies by mediating NOX2 (NADPH oxidase 2), SOD, GPX (Glutathione Peroxidase), and otherenzymes.

The combination of nanotechnology and medicine has promising applications. Nanocarrier-based drug delivery systems have attracted widespread interest with the rapid development of nanotechnology. Nanotechnology is used in the treatment of AS by increasing systemic drug circulation time, reducing off-target cytotoxicity of drugs, improving drug solubility, reducing the required dose, combining diagnostic and therapeutic drugs to form therapeutic drugs, and increasing the accumulation of drugs at specific sites [45]. In recent years, a variety of industrial nanoparticles (NPs) have been reported, including dendrimers, micelles, liposomes, and macrophage-biomimetic NPs for the targeted delivery of drugs [46–50], which focused on ROS scavenging strategies that effectively inhibit the formation of intraplaque foam cells [51]. Designing ROS-based NPs for treating AS is a promising therapeutic strategy. It allows the drug to reach specific lesions, prolonging the therapeutic effect, achieving targeted controlled release, and reducing adverse side effects [52, 53]. We reviewed the strategies for the treatment of atherosclerosis based on ROS-associated NPs in the microenvironment of atherosclerotic plaques, including ROS scavenging and modulating strategies, ROS responsive strategies, ROS responsive and ROS scavenging strategies, regulation of oxidative stress strategies by mediating enzymes (Fig. 1).

## ROS scavenging and modulating strategies based on NPs

### Inorganic NPs

The imbalance between ROS production and clearance leads to tissue and cellular dysfunction and accelerates the progression of AS. Therefore, drugs that effectively reduce ROS levels are essential for the treatment of AS (Table 1).

**Table 1:** ROS scavenging and modulating strategy

Cell model	Nanoparticles	Drug loading	ROS scavenging and modulating	Targeting strategies	Results	Refs.
Raw 264.7	CD9-HMSN@RSV	RSV	ROS generation inhibition	Active targeting (CD9)	Anti-aging, anti-inflammatory, targets macrophages	[56]
Raw 264.7	CFNs	\	Clear O_2_·^−^, ·OH	Active targeting (P-Selectin)	Anti-inflammatory, antioxidant, targeted plaques	[57]
Raw 264.7	IL10-cRGD-Lip	IL10	ROS generation inhibition	Active targeting (cRGD)	Anti-inflammatory, nitric oxide inhibition	[58]
Raw 264.7 HUVECs	PCZ@PB NCs	PB	Clear H_2_O_2_, O_2_·^−^	Passive targeting	Anti-inflammatory, antioxidant, and drug delivery	[59]
Raw 264.7 HUVECs VSMCs	HA-M@PB@ (PC + ART)	ART, PC	ROS generation inhibition	Passive targeting	Anti-inflammatory, lipid modulation, enhanced autophagy, antioxidant	[60]
Raw 264.7	Gd/CeO_2_ Nanozyme	\	Clear H_2_O_2_, O_2_·^−^	Passive targeting	Inhibition of foam cell formation, macrophage migration, broad spectrum ROS scavenging	[61]
Raw 264.7	Cur-MnO_2_/HA	Cur	Clear H_2_O_2_, O_2_·^−^, ·OH	Passive targeting	Inhibition of HIF-1α expression promotes macrophage polarization to M2 type	[62]
J774A.1 CHO	CeO_2_-Fe_3_O_4_@LDH	\	Clear H_2_O_2_	Passive targeting	The antioxidant, potential contrast agent	[55]
Raw 264.7 VSMCs	TPCD NPs	\	Clear H_2_O_2_, O_2_·^−^, ClO^−^	Passive targeting	Broad spectrum ROS scavenging, stabilized plaque	[63]
Raw 264.7	ATOR-loaded PLGA/miRNA	ATO	ROS generation inhibition	Passive targeting	Anti-inflammatory and promotes macrophage polarization to M2 type	[64]
Raw 264.7	TN-PdH	\	Clear H_2_O_2_, O_2_·^−^, ·OH	Passive targeting	Anti-inflammatory, antioxidant, induction of autophagy in macrophages	[65]

***Gd*** **Gadolinium;** ***Cur*** **Curcumin;** ***LDH*** **Layered double hydroxide;** ***TPCD*** **Tempol and a hydrogen-peroxide-eliminating compound of phenylboronic acid pinacol ester onto a cyclic polysaccharide β-cyclodextrin; ATOR Atorvastatin;** ***PLGA*** **Poly (lactic-co-glycolic acid);** ***CFNs*** **Fucoidan-chitosan nanoparticle; Lip Liposomes;** ***TN-PdH*** **A distinct tetrapod needle-like palladium-hydrogen;** ***MnO***_***2***_ **Manganese dioxide;** ***CeO***_***2***_ **Cerium dioxide;** ***Fe***_***3***_ ***O***_***4***_ **Triiron tetraoxide;** ***HA*** **Hyaluronic acid;** ***PC*** **Procyanidin;** ***ART*** **Artemisinin;** ***CZ NCs*** **Ceria-ZOL nanocomposites;** ***PB*** **Probucol;** ***HUVECs*** **Human umbilical vein endothelial cells;** ***RSV*** **Rosuvastatin**

In the past, the treatment of ROS-related diseases mainly relied on natural antioxidants, such as vitamin E, carotene, curcumin, and the results of clinical studies have shown that these natural antioxidants have not demonstrated satisfactory results [55]. In recent years, nanomedicines have been designed to be used in the treatment of ROS-related diseases. The study showed that CeO_2_ NPs exhibited good SOD mimetic activity, CAT mimetic activity, hydroxyl radical scavenging ability, and nitric oxide (NO) scavenging ability [54]. LDH is a two-dimensional layered nanomaterial that has been used as a carrier for drug delivery and bioimaging. Liu et al. constructed CeO_2_-Fe_3_O_4_@LDH nanocomposites by using the ROS scavenging effect of CeO_2_ and the molecular imaging technique of Fe_3_O_4_ NPs to attach both to the LDH surface by electrostatic interaction. The results showed that the CeO_2_-Fe_3_O_4_@LDH nanocomposite was successfully verified to be effective in scavenging ROS from macrophages by exploiting the antioxidant property of CeO_2_, and the MRI signal detection showed good therapeutic effects. The NPs can also be further modified to target ECs and VSMCs to scavenge ROS from various cells, providing a new therapeutic strategy for treating and monitoring AS [55]. The accumulation of ox-LDL in the intima is promoted by the excess ROS produced by macrophages and ECs. Nanosized enzymes with biomitetic characteristics of various enzymes (CAT, superoxide oxidase, etc.) can effectively scavenge intracellular and intra-tissue ROS and have great potential in the treatment of AS.

**Fig. 2:**
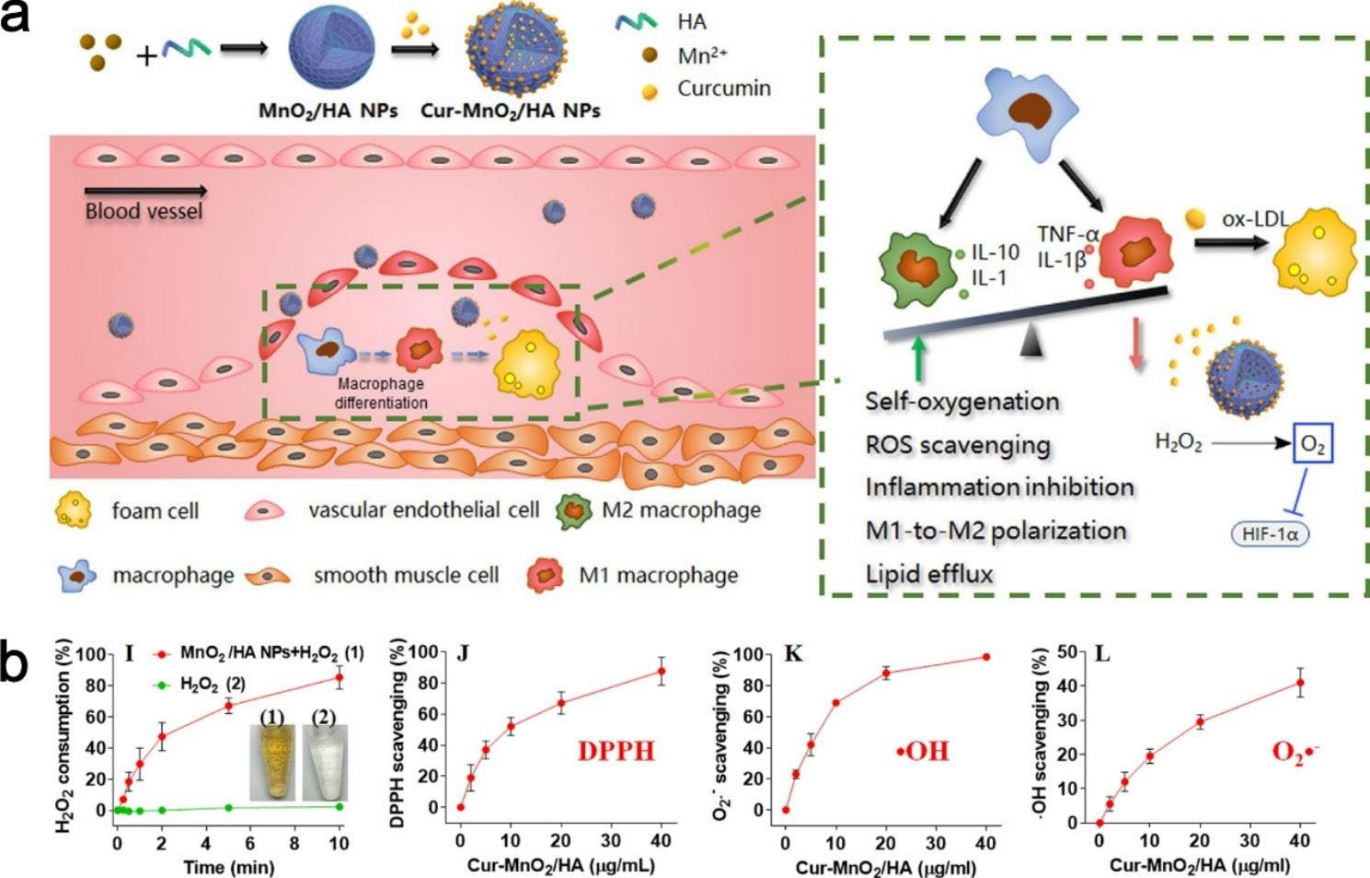
**a** Preparation of Cur-MnO_2_/HA and the therapeutic mechanism of AS. Cur-MnO_2_/HA effectively improves the intraplaque hypoxic environment through its own oxygen production, achieving anti-inflammation, ROS scavenging, promotion of macrophage polarization from M2 to M1 type, and lipid efflux. **b** Digestion of H_2_O_2_ and DPPH (2,2-diphenyl-1-picrylhydrazyl) scavenging activity, ·OH and O_2_^·−^ by Cur-MnO_2_/HA. Copyright 2022 by Sun [62]

Based on this, Gao et al. constructed metallo-gadolinium-doped Gd/CeO_2_ nanozymes and showed that the chemical doping of Gd promoted the ratio of Ce^3+^ on the surface of the nanozymes, thus enhancing the overall ROS scavenging ability. It showed that the Gd/CeO_2_ nanozymes effectively scavenged ROS at the cellular and histological levels and could alleviate oxidative stress induced by ox-LDL. Gd/CeO_2_ nanozymes significantly reduce vascular lesions by reducing lipid accumulation in macrophages and ECs and decreasing levels of inflammatory factors, such as IL-1, IL-6, IL-17, and tumor necrosis factor-alpha (TNF-α), ultimately inhibiting the progression of atherosclerotic lesions [61]. In addition, Gd/CeO_2_ can act as a T1-weighted MRI contrast agent that can produce sufficient contrast to differentiate the location of plaque during in vivo imaging, and the nanozymes hold promise as a potential diagnostic and therapeutic nanomedicine for ROS-induced AS. In general, internalized lipids from macrophages can be digested by transporters such as the ATP-binding cassette transporters A1 (ABCA1) and G1 (ABCG1) [66]. Extreme hypoxia is the pathological feature of the atherosclerotic plaque area, and the extreme hypoxia environment will up-regulate the expression of hypoxia-inducible factor 1α (HIF-1α). On the one hand, the up-regulation of HIF-1α expression can promote inflammation and the proliferation of smooth muscle cells and endothelial cells, and directly participate in the progress of atherosclerosis [56]. On the other hand, it will also inhibit cholesterol esterification and ABCA1 function, thus reducing cholesterol outflow [67, 68]. The imbalance between lipid input and efflux is disturbed, and the dysregulation of lipid metabolism promotes the polarization of macrophages to the M1 type, which further accelerates disease development. To address this dilemma, Sun et al. synthesized the Cur-MnO_2_/HA drug delivery system by piggybacking Cur onto MnO_2_/HA made from porous manganese dioxide and HA (Fig. 2).

The porous MnO_2_ not only greatly increases the drug loading capacity of Cur, but also generates oxygen, which can effectively alleviate the hypoxic microenvironment at the atherosclerotic plaque. Mediated by HA, the NPs can target the delivery of Cur to M1 macrophages with high CD44 expression. In this study, Cur-MnO_2_/HA NPs exhibited desirable ROS scavenging and anti-inflammatory capacity, and it were also able to promote the conversion of macrophages from M1 to M2 types. Furthermore, Cur-MnO_2_/HA NPs reduced lipid aggregation in macrophages and inhibited foam cell formation by modulating ABCA1. The NPs offer a promising therapeutic strategy to modulate the hypoxic microenvironment of AS through self-oxygenation and control the balance of lipid in-flow and out-flow to alleviate the disease [62].

### Organic NPs

Although inorganic NPs have a strong ability to scavenge ROS, their high in vivo toxicity, and hemolytic properties lead to poor biocompatibility, making the development of organic nanocarriers with ROS scavenging ability extremely important [69, 70]. Therefore, Leal et al. prepared PLGA NPs that can harbor both miRNA-124a and ATOR. PLGA NPs were functionalized with an antibody (anti-CD106) that allowed them to bind on VCAM-1 overexpressed in inflamed arterial endothelium. The NPs inhibited ROS production and expression of pro-inflammatory cytokines (IL-6, TNF-α) in macrophages and ECs while also inhibiting the uptake and transport of ox-LDL by macrophages and VSMCs. By co-carrying statins with miRNAs, synergistic treatment was achieved by exploiting their complementary characteristics, opening new doors for the treatment of AS [64]. The cell surface glycoprotein CD9, was strongly associated with aging and was highly expressed in atheromatous plaque areas [71–73]. and it could specifically bind to receptor on the surface of macrophages in the plaques. Therefore, Le et al. designed CD9-HMSN@RSV NPs capable of carrying the anti-aging drug rosuvastatin (RSV). The NPs were able to precisely target senescent vesicular macrophages, reducing ROS production, slowing down the senescence process, and down-regulating the secretion of TNF-α and IL-6. This may herald a more precise and effective therapeutic strategy for AS [56]. IL-10 is an important cytokine with unique anti-inflammatory properties [74]. In the available studies, it has been shown that IL-10 deficiency promotes the formation of AS [75]. In recent years, recombinant human IL-10 has been used in clinical trials such as inflammatory bowel disease, chronic hepatitis C, and rheumatoid arthritis [76, 77]. With its anti-inflammatory properties, it may help to inhibit the development of AS. Based on this, Li et al. designed IL-10-cRGD-Lip which can deliver IL-10 in a targeted way. Which reduced ROS levels in macrophages by 60% compared to the control group in vitro study. At the same time, it also reduced NO production by inhibiting the expression of the inflammatory factors IL-1β and TNF-α in macrophages, resulting in a reduction in the amount of nitrite produced by the reaction between NO and ROS, thus achieving cell protection [58]. ROS not only promotes apoptosis by activating NF-κB [78–80] but also disrupts redox-dependent signaling in the vascular wall, thereby contributing to the progression of atherosclerosis [81–83]. Therefore, the prevention of vascular oxidative stress and reduction of plaque production represents a rational strategy for atherosclerosis treatment. Wang et al. demonstrated that functional materials based on the esterification of the cyclic polysaccharide β-cyclodextrins (β-CD) with phenylboronic acid pinacol ester were able to eliminate hydrogen peroxide, showing anti-oxidative stress and anti-inflammatory activity. Based on this, the group developed a broad-spectrum ROS scavenging nanoparticle (TPCD) by covalently coupling a superoxide dismutase mimetic (Tempol) and phenylboronic acid pinacol ester to β-CD. In this study, TPCD was phagocytosed by macrophages and VSMCs and cleared excess intracellular ROS, attenuating ROS-induced inflammation and apoptosis. TPCD was also found to increase plaque stability by reducing macrophage infiltration and stromal metalloproteinase-9 (MMP-9) expression in plaques [63]. Polymeric nanocarriers can target drug delivery to atherosclerotic plaques but may affect the surrounding healthy environment or induce an inflammatory response due to their hydrolysis breakdown products [84]. Therefore, nanocarriers based on natural substances could be a suitable alternative. Fucoidan, a sulfated polysaccharide of marine origin, has a variety of favorable biological behaviors and is an excellent candidate for NPs development for AS treatment. Fucoidan has antioxidant properties and anti-inflammatory activity, inhibiting the formation of hydroxyl radicals and superoxide radicals. Liu et al. developed fucoidan-chitosan nanoparticles (CFNs) using non-toxic fucoidan. Since fucoidan can target p-Selectin, the NPs could block the recruitment and roll of leukocytes on platelets and endothelium by inhibiting p-Selectin. In this experiment, CFNs effectively scavenged hydroxyl radicals and superoxide radicals and inhibited the secretion of inflammatory factors IL-6, IL-1β, and TNF-α in macrophages. A large accumulation of green CFNs in atherosclerotic plaques was observed by fluorescence imaging, confirming their good targeting ability [57].

### Biomimetic NPs

Although inorganic and organic nano has a notable track record in the treatment of AS, its biological toxicity, immune phagocytosis and circulation time still limit its further development. The emergence of biomimetic NPs, therefore, provides us with new research directions. Fu et al. prepared biomimetic PCZ@PB NCs by encapsulating ceria-zoledronic acid nanocomposites (CZ NCs) equipped with probucol (PB) with platelet membranes. In vitro experiments showed that inflammatory macrophages were more likely to internalize PCZ@PB NCs than normal macrophages, which would facilitate intracellular ROS clearance. Secondly, PCZ@PB NCs could down-regulate the expression of two inflammatory factors, TNF-α and MMP-9, by inhibiting NF-κB, as a way to achieve anti-inflammatory effects. In vivo experiments, fluorescence showed that PCZ@PB NCs had more overlap with macrophage fluorescence signals at the plaques, which was attributed to the active targeting ability of platelet membranes [59]. As macrophages can be recruited to atherosclerotic plaques for targeting, erythrocyte membranes show significantly prolonged circulation time and improved drug accumulation in the lesioned region. Based on this, Zhou et al. designed a nano-complex HA-M@PB@(PC + ART) NPs encapsulated with macrophage and erythrocyte membranes and carrying artemisinin (ART) and procyanidin (PC). Their experiments revealed that HA-M@PB@(PC + ART) NPs reduce inflammation by inhibiting the RONS/NF-κB/NLRP3 pathway, thereby suppressing lipid influx. Secondly, HA-M@PB@(PC + ART) NPs enhance the AMPK/mTOR/autophagy pathway to promote cholesterol efflux. The membrane-modified nano complexes have good plaque targeting and immune escape capabilities, allowing them to circulate for longer periods, allowing more drugs to accumulate at the plaque, which will greatly enhance the therapeutic effect of AS [60]. The complex interplay between ROS signaling, inflammatory cytokines, and immune cells has led to the development of multifunctional nano-delivery systems that target these mechanisms more effectively to mitigate AS. Autophagy is the process of self-degradation of some cytoplasmic components in cells, such as misfolded or aggregated proteins, damaged organelles, and intracellular pathogens. Autophagy is important in maintaining intracellular homeostasis and suppressing inflammation, thereby contributing more to the clearance of ROS. Notably, macrophage autophagy is an effective strategy for the treatment of AS [85, 86]. Based on this, Hu et al. designed nanozymes that could load iron species onto a distinct tetrapod needle-like TN-PdH nanozyme and loaded the nanozyme with macrophages. The results showed that the nanozymes loaded with macrophages could target aggregation at plaques, the addition of iron species to the nanozymes allowed it to induce autophagy in macrophages, thus further enhancing the anti-inflammatory effect. This study guides the effective treatment of AS through a combination of ROS scavenging, hydrogen anti-inflammation, and autophagy activation [65].

## ROS responsive strategies for AS treatment

### Organic NPs

Inflammation participates in the development of AS widely, which is accompanied by the production of ROS, a family of ROS that generally includes H_2_O_2_, ·OH, O_2_·^−^, and HOCL [15]. ROS overproduction occurs in a wide range of diseases and emerging ROS-responsive (Table 2) materials have great potential for biomedical development [87].

**Table 2:** ROS responsive Strategy

Cell model	Nanoparticles	Drug loading	ROS response method	Targeting strategies	Results	Refs.
RAW 264.7	HASF@Cur	Cur	Material (Thioketal linkages and Ferrocene) has ROS responsiveness	Active targeting(CD44)	Lysosomal escape and reduction in plaque area	[88]
RAW 264.7	TPCDP@PMM	Pred	Material (Polymer blocks) has ROS responsiveness	Active targeting(VCAM-1, CD44)	Anti-inflammatory, hypolipidemic, diagnostic	[89]
RAW264.7 HUVECs	MM-AT-NPs	\	Material (Chitosan oligosaccharide)has ROS responsiveness	Passive targeting	Anti-inflammatory, immune escape	[48]
RAW264.7 HUVECs	MM/RNPs	RAP	Material (Boronic esters) has ROS responsiveness	Passive targeting	Immune escape, inhibition of macrophage, and smooth muscle cell proliferation	[90]
RAW 264.7 MAECs	HA-Fc/NP^3^_ST_	\	Material (Ferrocene) has ROS responsiveness ROS	Passive targeting	Anti-inflammatory, greater plaque penetration, and reduced plaque size	[91]

***HASF*** **Oligomeric hyaluronic acid-2’-[propane-2,2-diyllbls (thio)] diacetic acl-hydroxymethylferrocene;** ***HA-Fc*** **Hyaluronic acid-ferrocene;** ***NP*** ^***3***^_***ST***_ **Multivalent host-guest interactions between β-CD-anchored discoidal recombinant high-density lipoprotein;** ***MM*** **Macrophage membranes;** ***Pred*** **Prednisolone;** ***RAP*** **Rapamycin**

**Fig. 3:**
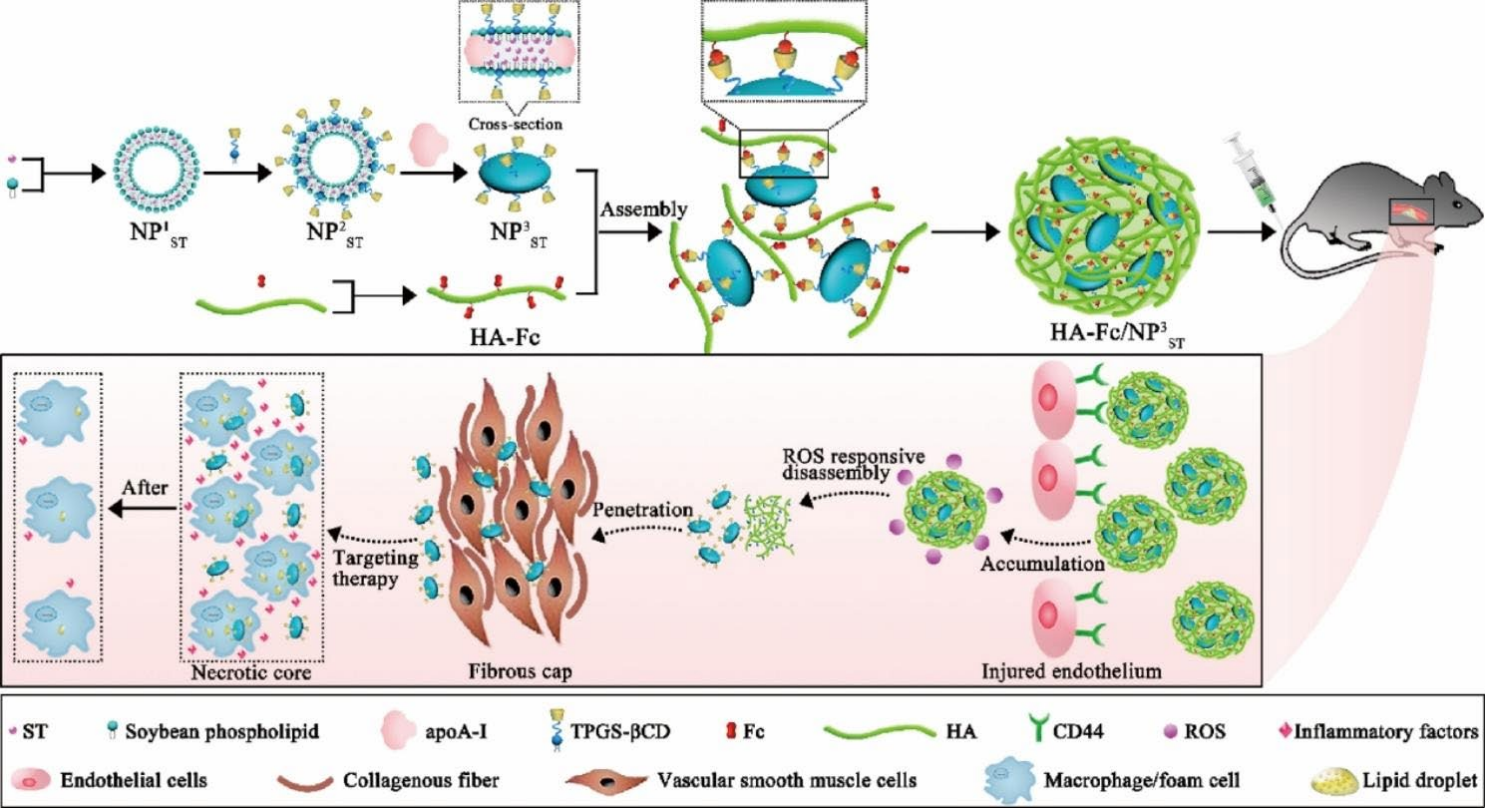
Schematic representation of the synthesis of HA-Fc/NP^3^_ST_ and the release of small-sized NP^3^_ST_ from HA-Fc/NP^3^_ST_ via the ROS response for anti-atherosclerotic treatment. Copyright 2023 by He [91]

Hou et al. synthesized HASF@Cur micelles by combining Cur with HASF, which can respond to ROS. In vitro assays, HASF@Cur micelles show H_2_O_2_ concentration-dependent release at H_2_O_2_ concentrations of less than 1 mM. The micelles were taken up by macrophages with favorable lysosomal escape ability. This allows the anti-inflammatory and antioxidant functions of Cur to be effectively performed [88]. NPs need to cross the fibrous cap of the plaque before reaching pathological macrophages/foam cells. Although particles in the 100–200 nm range are usually in the bloodstream for extended circulation times, their penetration in atherosclerotic plaques is relatively limited [92]. NPs below 40 nm can effectively penetrate plaque tissue and reach endothelial macrophages or foam cells [93]. A limited accumulation of the plaque due to its short circulation time limits its efficacy [94]. To overcome this challenge, He et al. designed variable-size HA-Fc/NP^3^_ST_ nano delivery systems. HA-Fc/NP^3^_ST_ targets aggregation at plaques by responding to CD44, and the accumulated HA-Fc/NP^3^_ST_ releases small-sized NP^3^_ST_ in response to ROS, which penetrates deep into the macrophage bulb and promotes macrophage cholesterol efflux (Fig. 3). Besides, HA-Fc/NP^3^_ST_ reduces the secretion of IL-6, TNF-α, and CCL2 inflammatory factors while promoting macrophage polarization to M2 type [91]. In addition to treating the three main pathological changes in endothelial damage, inflammatory response, and lipid deposition-nanoparticles with in vivo traceability can also be used for the identification and diagnosis of AS [95].

Ma et al. used supramolecular interaction to pack pred into β-CD and two-photon aggregation-induced luminescent active fluorophore cavities interconnected by ROS-responsive bonds and then wrapped with ROS-sensitive nano micelles to construct therapeutically integrated NPs (TPCDP@PMM). By tracking TPCDP@PMM in vivo, it was found to be significantly aggregated at the plaque, which would aid in the diagnosis of plaque. When TPCDP@PMM reaches the plaque site, in the presence of ROS and lipids, PMM disintegrates and TP separates from β-CD, releasing pred, thus achieving both lipid scavenging and anti-inflammatory therapeutic effects [89].

### 5.2 Biomimetic NPs

The immune functions of macrophages include phagocytosis, cytokine production, inflammatory activity, and antigen presentation. Macrophages have a dual role in processes such as tissue injury and inflammation: in inflammation-related diseases, macrophages are thought to exacerbate disease progression when inflammatory cells cannot be suppressed, conversely, macrophages can contribute to the clearance of inflammation by phagocytosing dead cells and being triggered into an M2-like macrophage state [96]. During the course of AS, macrophages are recruited to the lesion site and are involved in all stages of AS development [97–99]. Also, as an immune cells, macrophages have an innate immune escape function [100, 101]. Due to their excellent targeting and immune escape, biomimetic nano-particles prepared by macrophage membranes have been proven to be effective in the treatment of AS in many studies. Tang et al. synthesized MM/RNPs by camouflaging MM onto ROS-responsive NPs and loading them with rapamycin, which effectively evaded macrophage phagocytosis and prolonged blood circulation due to their biomimetic camouflage. At the same time, MM/RNPs were able to aggregate at inflammatory ECs through targeting with TNF-α. No embryonic death or developmental malformations were observed after the treatment of zebrafish embryos with MM/RNPs. This suggests that good biocompatibility could make it a long-term therapeutic agent for chronic vascular disease [90]. Gao et al. prepared an MM-AT-NPs drug delivery system by coating ROS-responsive NPs loaded with atorvastatin with MM. The macrophage membrane not only directed NPs to inflammatory tissues but also prevented NPs from being cleared from the reticuloendothelial system. Meanwhile, due to the presence of membrane antigens on macrophages (e.g. TNFR2, CD36, and CCR2), MM may effectively sequester key pro-inflammatory cytokines or chemokines to reduce their levels in the environment, thereby reducing local inflammation, and this synergistic effect of biomimetic drug delivery and inflammatory cytokine sequestration results in a significant enhancement of AS therapeutic efficacy [48].

### Nanoparticles-based ROS responsive and ROS scavenging for AS treatment strategies

#### Organic NPs

Inflammation and oxidative stress are the two main factors involved in the pathogenesis of AS. Due to the complexity of the microenvironment at the plaque, inflammation inhibition or reactive oxygen species scavenging treatments alone often fail to achieve the desired therapeutic effect. Therefore, the preparation of NPs with both ROS response and ROS scavenging capabilities to increase targeting ability and precise drug release is an effective way to enhance therapeutic efficacy (Table 3).

**Table 3:** ROS responsive and ROS scavenging strategies

Cell model	Nanoparticles	Drug loading	ROS scavenging	Targeting strategies	Results	Refs.	
RAW 264.7 HUVECs	LFP/PCDPD	Pred	Clear ROS	Active targeting (VCAM-1, CD44)	Anti-inflammatory, lipid removal, fluorescent imaging	[102]	
RAW 264.7	PLCDP@PMH	Pred	Clear ROS	Active targeting (VCAM-1, CD44)	Lipid modulation, inhibition of macrophage polarization to M1 type, plaque diagnosis	[103]	
RAW 264.7 LO2	HA/HPEMs	SIM	Clear ROS	Active targeting (CD44)	Anti-inflammatory, antioxidant, inhibits macrophage growth	[104]	
RAW 264.7	RBC/LFP@PMMP	Pred	Clear ROS	Passive targeting	Anti-inflammatory, antioxidant, and lipid imaging	[105]	
RAW 264.7 HUVECs	SV MC@RBCs	SIM	Clear ROS	Passive targeting	Anti-inflammatory, antioxidant, antithrombotic, shear stress responsive	[106]	
RAW 264.7 ECs	RP-PU	PB	Clear ROS	Passive targeting	Anti-inflammatory, antioxidant, hypolipidemic, inhibits foam cell formation	[107]	
RAW 264.7 HUVECs	KPF@MM-NPs	KPF	Clear ROS	Passive targeting	Anti-inflammatory, antioxidant, promotes macrophage polarization from M2 to M1 type and enhances internalization	[108]	
RAW 264.7 HUVECs	SA PAM@RBCs	SIM	Clear ROS	Passive targeting	Antioxidant reduces plaque count, shear stress responsive	[109]	
RAW 264.7	TPP@PMM	Pred	Clear ROS	Passive targeting	Anti-inflammatory, antioxidant, inflammatory diagnosis	[110]	
RAW 264.7 LO2	TPTS/C/T	SIM	Clear ROS	Passive targeting	Anti-inflammatory, antioxidant, inhibits macrophage polarization to M1 type, synergistic therapy	[111]	
RAW 264.7 HUVECs	SE-LNPs	SIM	Clear ROS	Passive targeting	Anti-inflammatory, antioxidant, anti-apoptotic, hypolipidemic, promotes macrophage polarization to M2 type	[112]	
RAW 264.7	PEG-PPS	Andro	Clear ROS	Passive targeting	Anti-inflammatory, antioxidant	[113]	

***TPP*** **Pred and two-photon fluorophore;** ***TPTS*** **Redoxresponsive nanoprodrug of simvastatin; SIM Simvastatin;** ***PEG-PPS*** **Poly(ethylene glycol) and poly(propylene sulphide);** ***Andro*** **Andrographolide;** ***RBC*** **Red blood cell;** ***SV MC*** **Mimvastatin-loaded micelles;** ***KPF*** **Kaempferol;** ***PAM*** **Cross-linked dendrimer**

**Fig. 4:**
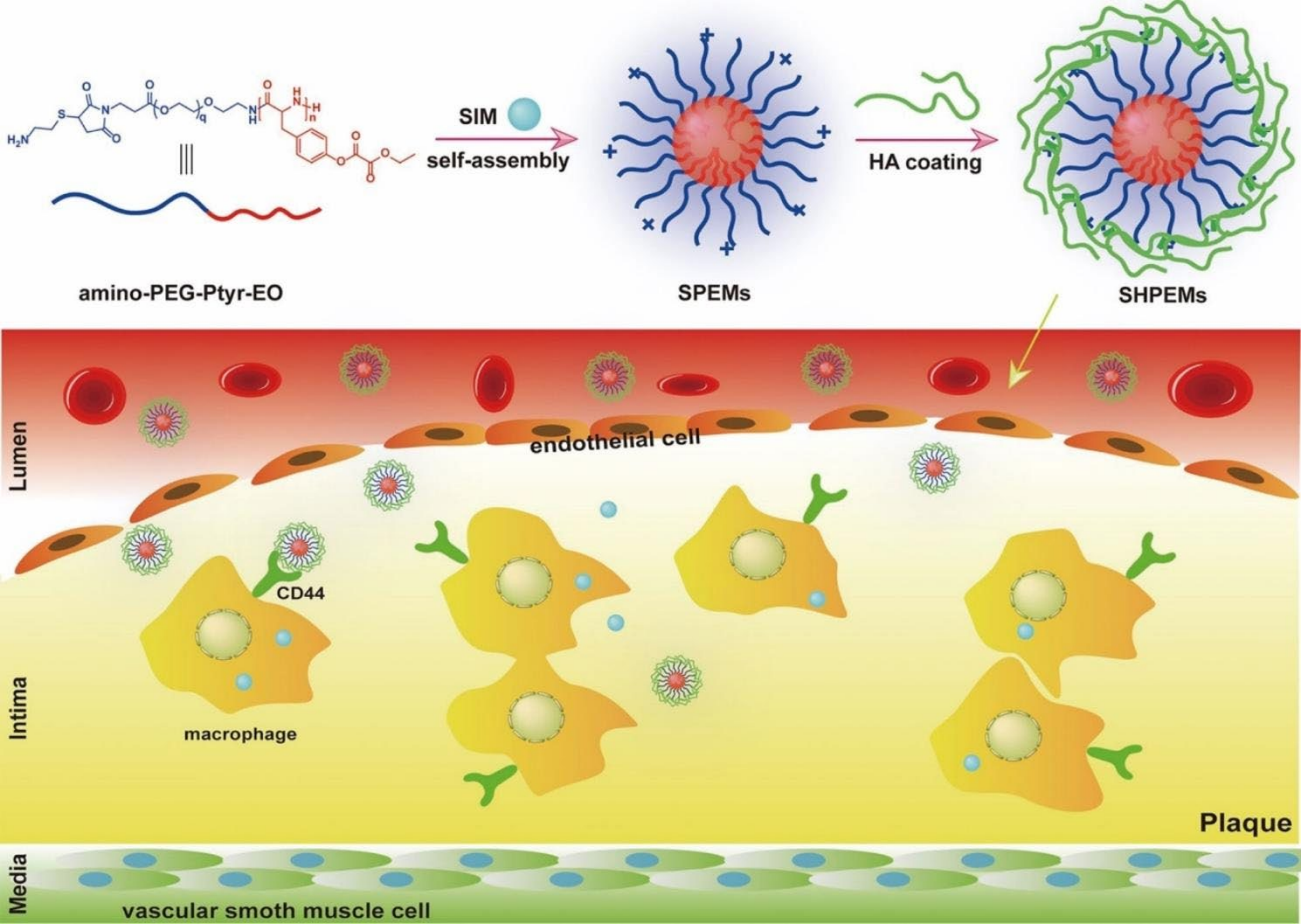
The diagram shows the preparation of SHPEMs and the targeting of SHPEMs across ECs to CD44 receptors on macrophages within the plaque, followed by nanoparticle disintegration for anti-inflammatory and ROS depletion purposes. Copyright 2020 by Mu [104]

The ROS response strategy also enables the controlled release of the drug and contributes to a sustained therapeutic effect. Wu et al. prepared polyethylene glycol and PEG-PPS nano micelles containing andrographolide, which rapidly release the encapsulated drug andrographolide in response to ROS and pathological sites to deplete ROS on its own, thereby effectively inhibiting the expression of pro-inflammatory cytokines and relieving oxidative stress [113]. Similarly, Zhao et al. synthesized ROS-responsive TPTS and developed TPTS/C/T, a fibronectin-targeted drug delivery system that can deliver both SIM and Tegretol, by exploiting the self-assembly behavior of TPTS. TPTS can release thiolated SIM by responding to high concentrations of ROS, followed by rehydration to release SIM. This experiment showed that TPTS and TPTS/C/T have good stability and can reduce off-target leakage of the drug. In addition, TPTS/C/T scavenged ROS and anti-inflammatory while also inhibiting macrophage polarisation to the M1 type [111]. CD44 is not only one of the specific biomarkers of macrophages but it is also upregulated and functionally activated on vascular endothelial, smooth muscle, and inflammatory cells in areas of inflammation [114]. In addition, CD44 regulates leukocyte adhesion, migration, and phenotype [115]. Mu et al. constructed micelles containing SIM using ROS-responsive and biodegradable poly (ethylene glycol)-poly (tyrosine-ethyl oxalate) (PEG-Ptyr-EO), then it was coated with HA to make it have the ability to target CD44 (Fig. 4). It has been shown that the micelles are on the one hand aggregated at the plaque through the targeting of CD44, on the other hand, they are enzymatically degradable and can consume ROS on their own at the pathological site, thus effectively inhibiting the accumulation of proinflammatory macrophages and reducing oxidative stress. Overall, these micelles with excellent targeting capabilities are an innovative option for the treatment of AS [104].

Early, rapid and accurate detection is the key to effective prevention and treatment of cardiovascular disease. The main imaging methods currently used to evaluate atherosclerotic plaques in small animals are CT, MRI, PET, and ultrasound imaging. With the development of bioengineering technology, more and more research teams are dedicated to the imaging of atherosclerotic plaques with NPs. Ma et al. bridged Pred with a two-photon fluorophore via a ROS-sensitive bond to form the diagnostic-therapeutic compound TPP, which was then assembled with the amphoteric polymer PMPC-PMEMA (PMM) to form the TPP@PMM micelles. The micelles release Pred in response to overexpressed ROS at the lesion site as a means of treatment. Using the features of TP fluorescence imaging to track TPP@PMM, it was observed that TPP@PMM can target aggregation at the plaque, and therefore the further diagnosis of plaque by TPP@PMM is a potential strategy [110]. In another study by the Ma, a three-in-one therapeutic complex was constructed and encapsulated into NPs called PLCDP@PMH together with a polymeric photoacoustic probe. PLCDP@PMH releases therapeutic complexes in response to high expression of ROS and MMP-9 at plaques, thereby inhibiting lipid uptake by reducing M1-type polarization of macrophages, while upregulating ABCA1 and ABCG1 expression to enhance lipid efflux and initiating β-CD for lipid clearance. In animal studies, PLCDP@PMH enables visual photoacoustic imaging with strong contrast compared to normal tissue, providing a new direction in the therapeutic diagnostics of early AS [103]. In addition to designing a rational treatment strategy, effective identification of plaque and targeted imaging of early lesions are of great importance in the diagnosis of AS. Therefore, Xu et al. constructed a multifunctional nanoparticle LFP/PCDPD with ROS-responsive drug release, lipid removal, and lipid-specific AIE fluorescence imaging. This study enables specific diagnosis of early AS and provides a new strategy for synergistic anti-inflammatory and lipid removal therapy [102].

In addition to micelles, organic nanoparticles also include polymeric NPs, liposomes, and high-density lipoprotein NPs[116]. Liposomes have been intensively studied since their discovery. Liposomes are valued for their unique composition and special physicochemical properties such as size and surface charge, and are considered to be good drug delivery carriers. At the same time, liposomes can reduce drug toxicity and prolong the duration of drug action by delaying renal metabolism and excretion [117, 118]. Wan et al. prepared liposomal NPs, SE-LNPs, which effectively inhibited ROS-induced apoptosis by releasing the antioxidant EGCG in response to H_2_O_2_ and thus achieved ROS scavenging. Moreover, their experimental results showed that SE-LNPs not only promoted the conversion of macrophages from M1 to M2 type, but also inhibited the expression of inflammatory factors TNF-α, IL-6, IL-1β, and monocyte chemoattractant protein-1 (MCP-1), which would greatly reduce the inflammatory response under this double guarantee [112].

### Biomimetic NPs

Compared to conventional delivery systems, biomimetic nano-delivery systems encapsulated in cell membranes allow for active targeting and targeted drug release [119]. Red blood cells (RBCs) are the most abundant cells in the blood. Mature RBCs have no nucleus or organelles and their large surface area is conducive to the loading of various drugs, making RBCs an ideal vehicle for drug delivery. At the same time, the proteins or polysaccharides on the surface of RBC membranes give them good biocompatibility and long circulation times. Therefore, RBCs are widely used in the study of biomimetic nano-drug delivery [120–122]. Liang et al. prepared biomimetic NPs RP-PU with ROS responsiveness using red blood cells and probucol. On the one hand, RP-PU significantly scavenged ROS and inhibited foam cell formation, on the other hand, it also inhibited the expression of ICAM-1 and MCP-1, reducing the adhesion of monocytes to ECs and delaying the formation of lesions [107]. In another study by Ma et al., they designed a biomimetic nano delivery platform (RBC/LFP@PMMP) to achieve long circulation, smaller drug leakage, and targeted plaques in vivo. Of note is the use of lipid-specific fluorophores (LFP) to enable lipid-specific bioimaging. In addition, the release of pred through the ROS response enables precise anti-inflammation. This diagnostic and therapeutic biomimetic nano-drug delivery platform will be a new therapeutic strategy for AS with great potential [105]. In addition to erythrocyte membranes, biomimetic NPs prepared using MM are one of the effective strategies for the treatment of AS. Using the targeting and immunological capabilities of macrophages, coating the surface of NPs with MM is a potential strategy to achieve long-term circulation and specific targeting of AS. Zhao et al. prepared a macrophage-mimicking delivery platform, KPF@MM-NPs, using KPF and MM. Using MM, KPF@MM-NPs can effectively localize and accumulate in atherosclerotic lesions. Their experiments revealed that KPF@MM-NPs inhibited the expression of the inflammatory factors p-p65 and p-IκB by blocking the ROS/NF-κB signaling pathway, while also repolarising macrophages from type M1 to type M2. In animal experiments, KPF@MM-NPs circulated significantly longer in ApoE-/- mice, the aortic plaque area was significantly reduced, and in their sections, macrophage infiltration was reduced and plaque stability was increased [108].

Acute cardiovascular disease (CVD) occurs when unstable atherosclerotic plaque ruptures inducing platelet aggregation and thrombosis due to vasoconstriction, predisposing to myocardial infarction and stroke, and is one of the leading causes of increased mortality and morbidity worldwide [123]. One of the many causes of plaque rupture is shear. The presence of atheromatous plaque narrows the lumen, which in turn leads to high shear forces at the plaque, and the high shear forces promote plaque rupture [124, 125]. Based on this, Shen et al. designed bionomic nano-SV MC@RBCs capable of responding to both ROS and high shear stress, which consisted of RBCs and SV MC. Experiments show that SV MC@RBCs release SV MC in response to high shear stress forces at plaques, followed by phagocytosis by macrophages, and then SV MC further exerts ROS scavenging and anti-inflammatory functions by responding to H_2_O_2_ in macrophages [106]. In another study by Shen, another dual-responsive biomimetic NPs SAPAM@RBCs was designed. SA PAM@RBCs separate SA PAM from RBCs by responding to high shear stress at the plaque, and SA PAM releases SIM in a concentration-dependent manner in response to H_2_O_2_ at the plaque and effectively removes H_2_O_2_ from macrophages. SA PAM also inhibits the uptake of ox-LDL by macrophages and reduces foam cell formation. Thus, a biomimetic delivery system with a dual response to ROS and shear stress would be a promising strategy for the treatment of AS [109].

### Nanoparticle-mediated therapeutic strategies for ROS-generating enzymes

ROS are produced by the electron transport chain in the mitochondria, on the one hand, and by several enzymes, mainly NOXs, XOD, eNOS, etc. [126]. Therefore, the researchers have used NPs to modulate the aforementioned enzymes, among others, to achieve anti-oxidative stress and ultimately the diagnosis or treatment of AS. Li et al. prepared a nanoparticle (HB-OLD7) that can target the delivery of siRNA to knock down NOX2 expression. In vivo tests, HB-OLD7 was injected intravenously into rats and a 30% reduction in NOX2 expression in the spleen and a significant reduction in ROS expression in rat carotid artery sections was observed. This study suggests that the knockdown of NOX2 significantly inhibited endothelial hyperplasia, which may be an effective strategy for the treatment of AS [127]. It has been shown in previous studies that NO has a protective effect on ECs [82]. Therefore, Imanparast et al. made mZD7349-SIM-PLGA-NPs using mZD7349 peptide, PLGA, and SIM. Compared with SIM-PLGA-NPs, mZD7349-SIM-PLGA-NPs treated HUVECs showed more phosphorylation of eNOS and increased NO bioavailability, thus promoting the repair of dysfunctional ECs. Further studies may be able to verify its potential anti-inflammatory and anti-atherosclerotic abilities, offering new possibilities for the treatment of AS [128]. Optimal selenium intake prevents AS by inhibiting inflammation, and oxidative stress, alleviating endothelial dysfunction, and preventing vascular apoptosis and calcification [129]. However, there is a narrow window of safe intake of selenium, and secondly, the final health outcomes of selenium supplementation vary widely depending on the type of supplementation [130, 131]. Selenium NPs have higher bioavailability, bioactivity, lower toxicity, and controlled release [132]. This opens up further possibilities for the further development of selenium. Guo et al. used selenium NPs (SeNPs) to study the mechanisms involved in the induction of AS in ApoE-/- mice by high cholesterol and high-fat diets. They found that 0.5 − 0.1 mM SeNPs were safe for cells, so treating ECs at this safe concentration, they observed that SeNPs inhibited H_2_O_2_-induced oxidative stress by upregulating the expression of SOD and GPX. In animal experiments, SeNPs were found to significantly alleviate hypercholesterolemia induced by high cholesterol and a high-fat diet by measuring the levels of TG, TC, and LDL-C in mice. Further sections of mouse aorta were stained and microscopically reduced proliferation of VSMCs and inhibition of foam cell production were observed. These results suggest that SeNPs may be a candidate drug for the treatment of AS [133].

### Prospects

Current anti-AS clinical agents include lipid-lowering agents (SIM, ATO, bezafibrate, fenofibrate, etc.), anti-platelet agents (aspirin, clopidogrel, prasugrel, etc.), antihypertensive agents (hydrochlorothiazide, metoprolol, cloxacillin, nifedipine, etc.) and the antioxidant drugs mentioned above. Although the traditional anti-atherosclerotic drugs are of clear benefit to the patient’s condition, they have particular shortcomings, such as significant systemic side effects, short circulation times, wide variations in individual efficacy, low bioavailability, and poor targeting ability.

Nanotechnology, on the other hand, is an effective means of solving these problems. The aggregation of NPs at the plaque can be divided into passive targeting strategies and active targeting strategies with ligand modification of the NPs.   Passive accumulation of NPs at plaques extravasation through leaky vasculature and subsequent immune cell sequestration (ELVIS effect), this is similar to the enhanced permeability and retention effect (EPR) in solid tumors. The difference in the mode of retention is the main difference between the two, with EPR resulting from abnormal tumor vasculature and lack of lymphatic drainage, and ELVIS being mediated by immune cells [134, 135]. Passive-targeted NPs can be divided into non-stimulus-responsive NPs and smart-responsive NPs based on their ability to respond to internal and external stimuli [135]. In the treatment of AS, non-stimulus-responsive NPs are mainly polymers such as PLGA, cyclodextrins, and chitosan [136]. In addition, smart response NPs have been developed by using the specific microenvironment of the plaque site (endogenous stimuli) or exogenous stimuli (for example, light, ultrasound, etc.) [137, 138]. Phototherapy is a relatively non-invasive and gentle treatment method that is gaining increasing attention in oncology treatment. It includes photothermal therapy (PTT) and photodynamic therapy (PDT). PTT uses a photosensitizer to convert near-infrared (NIR) light into heat, allowing precise control of the area of release in a dose- and time-dependent manner. Its non-invasive and highly selective nature has attracted many scientists in the biomedical field [139]. As a large number of pro-inflammatory M1 macrophages are present at the site of atherosclerotic disease and their secretion of large amounts of pro-inflammatory factors will lead to increased oxidative stress, the use of PTT to induce apoptosis of M1 macrophages can help to suppress inflammation and ROS, and this therapeutic strategy can be further explored in the future. In addition, when designing NPs based on a passive targeting strategy, various factors such as size, shape, and electrical potential need to be considered [140]. The variable-size NPs designed by He et al. above and the four-legged needle-like NPs designed by Hu et al. both demonstrated unique anti-atherosclerotic capabilities, proving that modifying the size and shape of the NPs by altering them is a highly promising therapeutic strategy for AS and therefore further pioneering in this area is valuable. In AS, the active targets are mainly divided into cellular (ECs, macrophages, VSMCs) and non-cellular targets. VCAM-1 and integrins on ECs, scavenger receptors on macrophages, and VSMCs are common biological targets, non-cellular components are mainly extracellular matrix components such as collagen. Active targeting of NPs with ligand modification, the most commonly used ligands are antibodies, peptides, polymers, etc., through which ligand modification can facilitate the selection of specific targets in cells or tissues, improve drug utilization and avoid systemic side effects [135]. Therefore, the active targeting strategy of ligand modification of NPs is one of the ways to effectively improve the targeting ability of NPs [141].

The vast majority of ROS in cells are of mitochondrial origin and therefore mitochondria play a key role in controlling ROS levels in the body. Mitochondria have a highly efficient antioxidant system. On the one hand, somatostatin in mitochondria isolates Fe^2+^ and thus prevents the Fenton reaction from occurring to induce hydroxyl generation [142, 143]. On the other hand, mitochondria scavenge ROS by relying on related antioxidant enzymes, such as superoxide dismutase that converts O_2_·^−^ to H_2_O_2_ [144]. H_2_O_2_ is then converted to H_2_O by CAT, peroxiredoxin, and GPX [17]. There is currently little research in academia on anti-AS therapy through the preparation of NPs that can modulate ROS production and ROS scavenging-related enzymes, and further exploration of this area may bring new hope for the treatment of AS.

In addition, the following issues should be considered to improve the translation rate of nanomedicines for the treatment of AS from the laboratory to the clinic. First, although synthetic NPs have been used extensively in the treatment of AS, their difficulty in replicating the full functionality of biological systems makes synthetic NPs susceptible to clearance by the immune system, lack of targeting ability, and toxic side effects. Biomimetic NPs have received increasing attention for their low toxicity, high biocompatibility, and good targeting ability [144]. Researchers have developed cell membrane-based NPs and effectively ameliorated oxidative stress in atherosclerosis, with the cell membrane and extracellular vesicle-based bionanotechnology strategies being the most common [119, 145, 146]. This provides a direction for nanomedicine researchers to explore the next generation of nanomedicine. Furthermore, as chronic inflammatory diseases such as inflammatory bowel disease (IBD) and rheumatoid arthritis (RA) share a disease microenvironment with AS, similar biologics can be drawn upon to treat diseases with similar pathophysiological features [147]. Modified therapeutic strategies for IBD and RA can be applied to AS. One study used macrophage microvesicles encapsulated with hollow manganese dioxide (H-MnO_2_) and DSP to prepare a messenger nanoenzyme (MMV-MnO_2_@DSP) with dual CAT and SOD-like enzymatic activity that selectively neutralizes inflammation-activated oxidative stress in macrophages, helps restore normal metabolic conversion of O_2_·^-^ and H_2_O_2_, and adjusts the intracellular balance of pro-/anti-inflammatory factors. It also inhibits the expression of TNF-α and IL-1β in inflammation-activated fibroblasts and synovial cells to block the feedback loop between the two in the RA microenvironment, effectively targeting the inflamed joints of RA rats and reducing synovitis and cartilage damage, and reprogramming the RA microenvironment through multi-targeted removal of ROS to achieve therapeutic improvement. This suggests that we can draw on the specific triggering mechanisms of extracellular vesicle-based nanoenzymes for other chronic diseases to develop therapeutic strategies that are more conducive to placing therapeutic agents in AS. Secondly, exosomes can be used as a natural carrier that can target plaque sites using their homing ability and carry a variety of active molecules (for example, RNA, non-coding small RNAs, proteins, etc.) on their own and are therefore touted as the next generation of gene carriers [148]. As exosomes themselves can also act as therapeutic substances, the treatment of AS based on extracellular vesicles is a very promising cell-free therapeutic strategy for the future. Finally, most nanomedicines for the treatment of AS are complex to prepare. The manufacturing process and cost of mass production should be carefully considered when developing them into AS nano drugs for clinical applications. Nanomaterials with inherent imaging and therapeutic properties can be used as multifunctional nanomedicines to minimize manufacturing processes and costs. Thus, there is still much room for improvement in AS nano drugs to provide relief with minimal side effects for all patients.

### Challenge

In general, the development of ROS clearance strategy nanotechnology is promising for the precise treatment of AS, but there are still shortcomings. Firstly, the fabrication of NPs is complex and quality control is difficult, secondly, the construction of nanotherapeutic systems is more complicated and biosafety needs more experiments to verify. In addition, its long-term side effects, in vivo metabolic pathways, and drug delivery methods need to be further investigated. Therefore, it is particularly important to develop a safe, simple, and efficient integrated platform for nano-therapy. Further individualization and effective translation to the clinic will be the main challenge ahead.

## Data Availability

Not applicable.

## Abbreviations

AS: Atherosclerosis
ABCA1: ATP-binding cassette transporters A1
ABCG1: ATP-binding cassette transporters G1
ART: Artemisinin
ATOR: Atorvastatin
Andro: Andrographolide
β-CD: β-cyclodextrins
CAT: Catalase
CeO_2_: Cerium dioxide
CFNs: Fucoidan-chitosan nanoparticle
Cur: Curcumin
CVD: Cardiovascular disease
CZ NCs: Ceria-ZOL nanocomposites
DPPH: 2,2-diphenyl-1-picrylhydrazyl
ECs: Endothelial cells
ELVIS effect: Extravasation through leaky vasculature and subsequent immune cell sequestration
EPRE: Enhanced permeability and retention effect
Fe_3_O_4_: Triiron tetraoxide
Gd: Gadolinium
GPX: Glutathione peroxidase
GST: Glutathione-S-transferase
HA: Hyaluronic acid
HASF: Oligomeric hyaluronic acid-2′-[propane-2,2-diyllbls (thio)] diacetic acl-hydroxymethylferrocene
HA-Fc: Hyaluronic acid-ferrocene
H_2_O_2_: Hydrogen peroxide
HIF-1α: Hypoxia-inducible factor 1α
HOCL: Hypochlorous acid
HUVECs: Human umbilical vein endothelial cells
IBD: Inflammatory bowel disease
IL: Interleukin
KPF: Kaempferol
LDL: Low-density lipoprotein
LDH: Layered double hydroxide
Lip: Liposomes
LFP: Lipid-specific fluorophores
MCP-1: Monocyte chemoattractant protein-1
MM: Macrophage membranes
MMP-9: Metalloproteinase-9
MnO_2_: Manganese dioxide
NPs: Nanoparticles
NADPH: Nicotinamide adenine dinucleotide phosphate
NOXs: Nicotinamide adenine dinucleotide phosphate oxidases
NOX2: NADPH oxidase 2
NO: Nitric oxide
NOS: Nitric oxide synthase
NIR: Near-infrared
NP^3^_ST_: Multivalent host-guest interactions between β-CD-anchored discoidal recombinant high-density lipoprotein
NF-κB: NF-kappaB
O_2_·^−^: Superoxide anion
·OH: Hydroxyl radical
O_3_: Ozone
ox-LDL: Oxidized low- density lipoprotein
PAM: Cross-linked dendrimer
PB: Probucol
PC: Procyanidin
PEG-PPS: Poly (ethylene glycol) and poly (propylene sulphide)
PLGA: Poly (lactic-co-glycolic acid)
POD: Peroxidase
PTT: Photothermal therapy
PDT: Photodynamic therapy
Pred: Prednisolone
ROS: Reactive oxygen species
ROO·: Peroxyl radical
RAP: Rapamycin
RBCs: Red blood cells
RA: Rheumatoid arthritis
SV MC: Simvastatin-loaded micelles
SIM: Simvastatin
SOD: Superoxide dismutase
RSV: Rosuvastatin
TPTS: Redoxresponsive nanoprodrug of simvastatin
TPP: Pred and two-photon fluorophore
TPCD: Tempol and a hydrogen-peroxide-eliminating compound of phenylboronic acid pinacol ester onto a cyclic polysaccharide β-cyclodextrin
TNF-α: Tumor necrosis factor-alpha
TN-PdH: A distinct tetrapod needle-like palladium-hydrogen
VCAM-1: Vascular cellular adhesion molecule-1
VSMCs: Vascular smooth muscle cells
XOD: Xanthine oxidase
